# *Saussurea lappa* Ethanolic Extract Attenuates Triamcinolone Acetonide-Induced Pulmonary and Splenic Tissue Damage in Rats via Modulation of Oxidative Stress, Inflammation, and Apoptosis

**DOI:** 10.3390/antiox9050396

**Published:** 2020-05-08

**Authors:** Ghada I. Abd El-Rahman, Amany Behairy, Nora M. Elseddawy, Gaber El-Saber Batiha, Wael N. Hozzein, Dina M. Khodeer, Yasmina M. Abd-Elhakim

**Affiliations:** 1 Department of Clinical Pathology, Faculty of Veterinary medicine, Zagazig University, Zagazig 44519, Egypt; gana660@gmail.com; 2 Department of Physiology, Faculty of Veterinary Medicine, Zagazig University, Zagazig 44519, Egypt; amanybehairy25688@gmail.com; 3 Department of Pathology, Faculty of Veterinary medicine, Zagazig University, Zagazig 44519 Egypt; noura.elseddawy@yahoo.com; 4 Department of Pharmacology and Therapeutics, Faculty of Veterinary Medicine, Damanhour University, Damanhour 22511, AlBeheira, Egypt; gaberbatiha@gmail.com; 5 National Research Center for Protozoan Diseases, Obihiro University of Agriculture and Veterinary Medicine, Nishi 2-13, Inada-cho, Obihiro, Hokkaido 080-8555, Japan; 6 Bioproducts Research Chair, Zoology Department, College of Science, King Saud University, Riyadh 11451, Saudi Arabia; whozzein@ksu.edu.sa; 7 Botany and Microbiology Department, Faculty of Science, Beni-Suef University, Beni-Suef 62511, Egypt; 8 Department of Pharmacology and Toxicology, Faculty of Pharmacy, Suez Canal University, Ismailia 41522, Egypt; dina_khoudaer@pharm.suez.edu.eg; 9 Department of Forensic Medicine and Toxicology, Faculty of Veterinary Medicine, Zagazig University, Zagazig 44519, Egypt

**Keywords:** *Saussurea lappa*, oxidative stress, inflammation, caspase-3, triamcinolone acetonide, CD8^+^

## Abstract

*Background:* In this era, worldwide interest has been directed towards using natural antioxidants to guard against drug side effects. *Saussurea lappa* is a famous medicinal plant with many biologically active compounds. Triamcinolone acetonide (TA) is an extensively used glucocorticoid. Hence, this study explored, for the first time, the possible beneficial effects of *S. lappa* ethanolic extract on TA-induced oxidative damage in the lung and spleen of rats. *Methods*: Five experimental groups were used: control group, *S. lappa*-treated group (600 mg/kg/day, orally), TA-treated group (40 mg/kg/twice/week I/P), *S. lappa* + TA co-treated group, and *S. lappa*/TA prophylactic group. *Results:* TA exposure significantly induced leukocytosis and neutrophilia. In addition, TA significantly reduced the levels of C-reactive protein, interleukin-12, tumor necrosis factor α, and immunoglobulins. Lung Caspase-3 overexpression and splenic CD8^+^ downregulation were also noted in the TA group. TA treatment significantly increased malondialdehyde concentration but reduced superoxide dismutase and glutathione peroxidase activities. *S. lappa* counteracted the TA oxidative and apoptotic effects. The best results were recorded in the prophylactic group. *Conclusions:* *S. lappa* has a remarkable protective effect via its anti-inflammatory, anti-apoptotic, and antioxidant capacity. Thus, it could be a candidate as a natural antioxidant to face glucocorticoid’s harmful side effects.

## 1. Introduction

Recently, various studies have highlighted the implication of oxidative stress in adverse side effects associated with drug use [1,2]. Hence, a global trend has focused on incorporating natural antioxidants as strategies to mitigate the side effects of drug use [3,4]. Herbal medicines can modulate many functions including free radical release, histamine release, phagocytosis, and cytokine and immunoglobulin secretion with the regulation of cell receptors and lymphocyte expression [5,6,7]. One of the widely used medicinal plants is *Saussurea lappa* (*S. lappa*), family *Asteraceae*, which is commonly known as Costus. Sesquiterpene lactones such as costunolide, dehydrocostus lactone, and cynaropicrin are the main constituents of *S. lappa* [8]. *S. lappa* can be utilized as an elective antioxidant operator in both the medical and food industries [9] via scavenging of nitric oxide (NO), 2,2-diphenyl-1-picrylhydrazyl, and superoxide radicals with lipid peroxidation inhibition [10]. *S. lappa* roots have been extensively suggested for treatment of inflammation-associated ailments such as asthma, chronic gastritis, rheumatoid arthritis, and bronchitis [11]. The anti-inflammatory action of the sesquiterpene lactone fraction of *S. lappa* may be related to the maintenance of lysosomal membranes with an antiproliferative consequence [12]. Cynaropicrin in the inflammatory response inhibited tumor necrosis factor-alpha (TNF-α) and NO and proliferated lymphocytes CD4^+^ and CD8^+^ via conjugation with sulfhydryl groups of target proteins [13].

Various health disorders have been associated with drugs and xenobiotics that exist in the human environment [2,14]. Glucocorticoids (GCs) are one of the most commonly prescribed drugs all over the world due to the great number of patients treated and the variability of medical applications [15]. GCs are primary stress hormones that cross cell membranes and exert their physiological roles by promoting glucocorticoid receptor action [16]. Triamcinolone acetonide (TA) is an effective synthetic GC, effectively used to treat retinal vein occlusion, rhinitis, uveitis, and diabetic macular edema [17,18]. At high doses, TA also can be used in the treatment of osteoarthritis, Addison’s disease, rheumatoid arthritis, leukemia, and hypersensitivity [19]. Additionally, TA is widely used to treat inflammation in dogs and cats [20] and performance-related injuries in horses [21]. TA showed very potent anti-inflammatory and analgesic activity in both osteochondral fragmentation [22] and acute lipopolysaccharide-induced lameness models [23].

The anti-inflammatory activity of TA could be through the inhibition of the number of basal cells in the blood [24]. Additionally, TA immunosuppressive action is related to inhibition of the appearance of some surface human leukocyte antigens and cluster of differentiation (CD) antigens of dendritic cells [25]. Despite the broad therapeutic uses of TA, its poor solubility in aqueous solutions results in slow penetration and diffusion to the tissue and cellular layers, causing longstanding and deleterious effects [26]. Regarding the immune response, TA reduced circulating antibody titers, splenic antibody-producing cells, and splenic lymphocyte numbers in fish immunized to *Yersinia ruckeri* [27]. Human CD8^+^ T cells showed less sensitivity to GC-mediated inhibition of mitogen-induced proliferation than CD4^+^ T cells [28]. Chung et al. [29] reported the increased reactive oxygen species (ROS) output inside TA-treated retinal cells compared with dexamethasone-treated and control cells. Several agents, which are considered oxidants or inducers of cellular oxidative metabolism, are implicated in cell death incidence, including apoptosis [30]. Repeated administration of TA decreased cell viability and caused permanent damage to cells [31].

Caspases are protease enzymes, which belong to the cysteine-aspartic acid endo-peptidases family [32]. Caspases are found mostly in the cytoplasm as inactive enzyme precursors or zymogens. When caspases are activated, serious irreversible alterations occur in the biochemical constituents and the morphology of cells [33]. Caspase-3 is the most invigorated caspase in apoptotic cells, indicating its pivotal role in the programmed cell death process [34]. Caspase-3 has been implicated as the key caspase effector in GC-mediated apoptosis [35]. It has been found that the apoptosis in mouse airway epithelial cells was stimulated by a high dexamethasone dose in normal and inflamed cells [36].

Despite the earlier reported biological activities of *S. lappa*, its potency to counteract the TA-induced immunosuppression and oxidative damage of the lungs has not yet been studied. Little is known about the modulatory role of *S. lappa* on TA anti-inflammatory activity. In addition, apoptotic activities of the active components of *S. lappa* have been responsible for anticancer activity in cancer cell lines, but little is known about the apoptotic effect of *S. lappa* on normal cells. Hence, this study was designed to assess the effect of *S. lappa* ethanolic extract on hematological indices, immunoglobulin levels, and pro-inflammatory cytokines in TA-treated rats. In addition, oxidative stress and lipid peroxidation indices were evaluated. Additionally, the probable underlying mechanisms were investigated by assessing Caspase-3 and CD8^+^ immune expression in lung and spleen tissues, respectively. Furthermore, the study compared the prophylactic and therapeutic potency of *S. lappa* ethanolic extract.

## 2. Materials and Methods

### 2.1. Plant Material Extraction

*S. lappa* roots were purchased from a herbal store in Sharkia governorate, Egypt. The plant was distinguished and verified within the Botany department at Zagazig University (Zagazig, Egypt). Whole plants were air-dried, processed into a moderately coarse powder, and kept in tightly closed bottles for later use in the extraction procedure. About 100 g of the obtained powder was soaked in 1000 mL of ethanol (70%) at room temperature and soaked for three days. The filtration of this solution occured through Whatman grade-1 filter paper in a funnel under vacuum. After that, the filtrate was subjected to the rotary evaporator to dry and evaporate the liquid under reduced pressure. Crude ethanolic extract of *S. lappa* roots was collected, which was further lyophilized to a dry powder [37]. Finally, the samples were stored at 4 °C until use.

### 2.2. Gas Chromatography/Mass Spectrometry Analysis (GC–MS) of S. lappa Ethanol Extract

A GC-ISQ mass spectrometer (Thermo Scientific, Austin, TX, USA) with a direct capillary column TG–5MS (30 m × 0.25 mm × 0.25 µm film thickness) was used for characterization of the components of the *S. lappa* ethanol extract sample. The temperature of the column oven was first held at 55 °C and then raised by 5 °C/min to 250 °C, held for 2 min, then increased to 300 °C at 25 °C/min. The temperature of the injector was kept at 270 °C. A carrier gas of helium was used at a constant flow rate of 1 mL/min. The solvent interval was 4 min, and 1 µL diluted samples were automatically injected by AS3000 Autosampler coupled with GC in the split mode. Electron ionization mass spectra were composed at 70 eV ionization voltages over the range of *m/z* 50–650 in full scan mode. The transfer line and ion source were set at 280 °C and 200 °C, respectively. The components were recognized by matching their mass spectra and retention times with those of the NIST14 and WILEY 09 mass spectral databases.

### 2.3. Tested Drugs, Chemicals, and Reagents

TA was obtained in a commercial form (Epirelefan vial of 1 mL sterile aqueous suspension) that had 40 mg of TA/mL. It is distributed by Egyptian International Pharmaceutical Industries Co. (EIPICO, 10th of Ramadan, Egypt). Commercial enzyme-linked immunosorbent assays (ELISA) kits (Spinreact, Girona, Spain) were adopted to estimate IgM, IgG, and C-reactive protein (CRP) (Biosciences, Dun Laoghaire, Co Dublin, Ireland). All other chemicals/reagents used were of analytical grade and purchased from (Sigma, St. Louis, MO, USA).

### 2.4. Animal Grouping and Experimental Design

Forty adult male albino rats weighing 150–160 g were acquired from the Laboratory Animal Unit of Veterinary Medicine, Zagazig University, Egypt. Rats were housed in metal cages and kept at room temperature (25±5 °C) with a normal 12 h light/12 h dark cycle. They were fed a typical pellet diet with water ad libitum and adapted to the laboratory conditions for seven days before the experiment started. The Institutional Animal Care and Use Committee of Zagazig University approved the present protocol with the reference number ZU-IACUC/2/F/37/2020.

**Figure 1:**
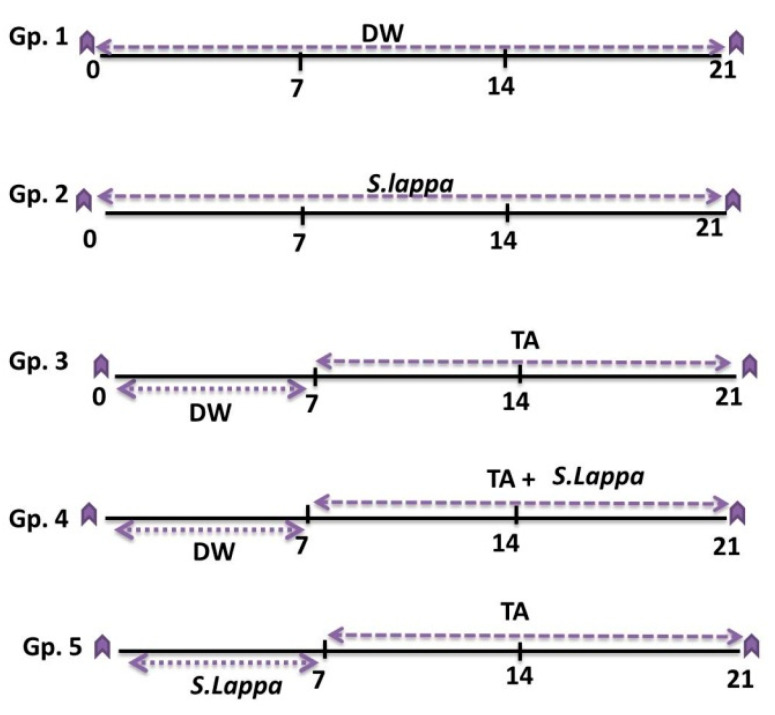
Experimental groups and treatments: *Saussurea lappa* ethanolic extract (*S. lappa*; 600 mg/kg body weight, orally via a gastric tube), triamcinolone acetonide (TA; 40 mg/kg twice a week intraperitoneally), and distilled water (DW; 1 mL/rat orally).

As described in Figure 1, the rats were randomly allocated into five groups of eight rats each as follows:

- Gp. 1 (control): Each rat was orally administered 1 ml distilled water for three successive weeks.
- Gp. 2 (*S. lappa*): Each rat orally received 600 mg *S. lappa* ethanolic extract/kg daily via a gastric tube [10] for three weeks.
- Gp. 3 (TA): Each rat was orally administered distilled water (1 ml/rat) for one week, then intraperitoneally injected with 40 mg/kg of TA twice a week for two weeks [38].
- Gp. 4 (co-treated): Each rat was orally administered distilled water (1 mL/rat) for one week, then concurrently treated with TA and *S. lappa* extract for two weeks by the same previous doses and routes.
- Gp. 5 (prophylactic group): Each rat received *S. lappa* extract for one week, and then was concurrently treated with TA and *S. lappa* extract for two weeks by the same previous doses and routes. Rats were given their *S. Lappa* dose via gastric gavage needle in 1 mL distilled water.

The body weights of all rats in each group were recorded at the start and end of the experiment. Calculation of the spleen and lung indexes were made after sacrificing the animals according to this formula: organ index (mg/g) = organ weight (mg) / body weight (g) [39].

### 2.5. Blood and Tissue Sampling

After the end of the experiment, blood samples were obtained from the retro-orbital venous plexus of anesthetized rats and were drained into sterile ethylenediaminetetraacetic acid (EDTA)-tubes for hematological analysis. Another blood sample was collected into a sterile centrifuge tube devoid of anticoagulant for serum isolation for the biochemical assay. After sacrificing the animals, spleen and lung specimens were quickly collected and partitioned into two parcels. The 1st parcel was kept at −20 °C until used for determining the oxidant and antioxidant biomarkers. The 2nd portion was preserved in 10% neutral formalin for H&E histopathological and immunohistochemical investigation of lung Caspase-3 and spleen CD8^+^.

### 2.6. Hematological Studies

The complete blood count (erythrogam and leukogram picture) was determined by an automated blood cell analyzer (Hemascreen 18, Hospitex Diagnostic, Calenzano, Italy) [40].

### 2.7. Analysis of Inflammatory Markers and Immunoglobulins Level

Serum IL-12 and TNF-α were estimated by a commercial quantitative ELISA measurement (MyBioSource, San Diego, CA, USA) based on Grassi et al. [41] and Beutler et al. [42], respectively. In addition, the concentration of serum CRP was measured using ELISA kits of [43]. Humoral immune response indicators (serum IgG and IgM) were evaluated by a specific Rat IgG and IgM ELISA kit following the method of [44]. All reading values were obtained using a plate reader and a standard curve.

### 2.8. Screening of Lipid Peroxidation and Antioxidant Enzymes

Malondialdehyde (MDA) concentration and superoxide dismutase (SOD) and glutathione peroxidase (GPx) activities were measured in the splenic and lung tissues by ELISA kits of Cusabio Biotech. Co., Ltd. (Houston, TX, USA), using the methods depicted by Kakkar et al. [45], Paglia and Valentine [46], and Ohkawa et al. [47], respectively.

### 2.9. Histopathological Investigation

Specimens of spleen and lung were collected from different groups. The collected specimens were fixed in 10% buffered neutral formalin solution, dehydrated in gradual alcohol (70%–100%), cleared in xylene, and implanted in paraffin. Then, five-micron-thick paraffin sections were prepared and stained with hematoxylin and eosin (H&E) dyes and then inspected microscopically [48].

### 2.10. Immuno-Histochemical Technique

Spleen and lung specimens were prepared for immunohistochemistry using the avidin-biotin- peroxidase (ABC) method [49]. To deactivate endogenous peroxidases, the sections were exposed to 3% hydrogen peroxide for 10 min after deparaffinization. The deparaffinized sections were heated in 10 mM citrate buffer at 121 °C for 30 min then blocked in 5% goat serum for 20 min. After that, they were incubated with a primary monoclonal antibody specific for spleen CD8^+^ and lung Caspase 3 (MCA1768, Serotec, Kidlington, UK) (AB-20074b, Sangon Biotech, Shanghai, China), respectively, overnight at 4 °C. Sections were incubated with a biotin-conjugated secondary antibody (1:2000 in phosphate buffer saline (PBS); Cat. No. sc-2040) for 20 min at 32 °C after three washes with PBS, then incubated with horseradish peroxidase (HRP)-labelled streptavidin. The antibody binding was determined using a substrate/chromogen (diaminobenzidine) reagent and Mayer’s hematoxylin. The cellular reaction appeared as brown granules inside the nucleus of epithelial cells, the staining intensity of the samples was evaluated as mild, moderate, or strong and scored by calculating the positive cells/image percentage by a 5-point scale as follows: + = < 10% (negative to weak expression), ++ = 10%–25% (mild expression), +++ = 26%–50% (moderate expression), ++++ = 51%–75% (strong expression).

### 2.11. Statistical Analysis

All data were expressed as the mean ± standard error (SE) and were statistically analyzed using one-way ANOVA followed by Duncan’s posthoc test for multiple comparisons, with the help of the SPSS software program (SPSS 16.0, IBM Corp. Armonk, NY, USA). The significant differences were detected at (*p* < 0.05), *n* = 5/group.

## 3. Results

### 3.1. GC-MS Profile of S. lappa Ethanolic Extract

**Figure 2:**
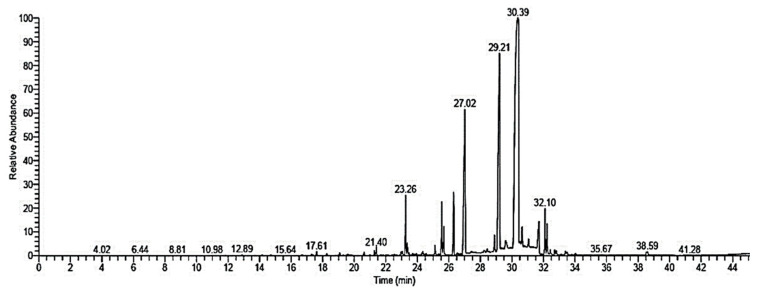
GC-MS chromatogram of *Saussurea lappa* ethanolic extract.

The *S. lappa* ethanolic extract GC-MS analysis showed the main components together with their retention times and relative percentage of the total peak area (Figure 2 and Table 1). The data demonstrated that the extract contained 26 components, which were dominated by dehydrocostuslactone (49.68%), dihydrodehydrocostus lactone (16.34%), caryophyllene oxide (11.03%), saussurea lactone (4.16%), costunolide (3.59%), and beta-costol (2.74%).

### 3.2. Body and Organ Weight Changes

Estimation of the bodyweight at the start and after the end of the experiment showed a significant increase (*p* < 0.05) in the weights of the *S. lappa*-treated group compared to the control group (Table 2). However, after giving TA, there was a significant reduction in the final body weight and body weight gain, together with spleen and lung indexes relative to those of normal control. However, such decreases in body weights and organ indexes were significantly rescued after *S. lappa* administration to TA-exposed animals compared to only TA-treated rats. Notably, more significant recovery (*p* < 0.05) was detected in the prophylactic group.

### 3.3. Hematological Findings

Table 3 demonstrates the changes in the erythrogram and leukogram components in rats orally administered *S. lappa* before or with TA injection.

Initially, no significant difference in the erythorgram elements including red blood cells (RBCs), hemoglobulin (Hb), and packed cell volume (PCV) in the *S. lappa*-treated group relative to the control group was found. On the other hand, a significant (*p* < 0.05) elevation in the RBCs count, Hb concentration, and PCV level were recorded following TA injection compared to control rats. However, *S. lappa* treatment either in combination with TA or as a prophylactic dose minimized the elevation of erythrogram elements evoked via a TA challenge in a significant manner (*p* < 0.05), which was more prominent in the protective group.

Concerning leukogram findings, a significant increase in the total leukocytic count (TLC) concomitant with lymphocytosis was apparent in the *S. lappa*-treated group relative to the control group. However, no significant changes in the counts of other leukocyte types were recorded. On the other hand, significant (*p* < 0.05) leukocytosis and neutrophilia were obvious in the TA-exposed group compared to the control group. However, a significant reduction in the count of lymphocytes, monocytes, eosinophils, and basophils was evident in the TA-exposed group compared to control rats. Meanwhile, the *S. lappa* use with TA in both prophylactic and treated groups resulted in a significant enhancement in these indicators in comparison with the TA-only-exposed group.

### 3.4. Inflammatory and Immunological Markers

**Figure 3:**
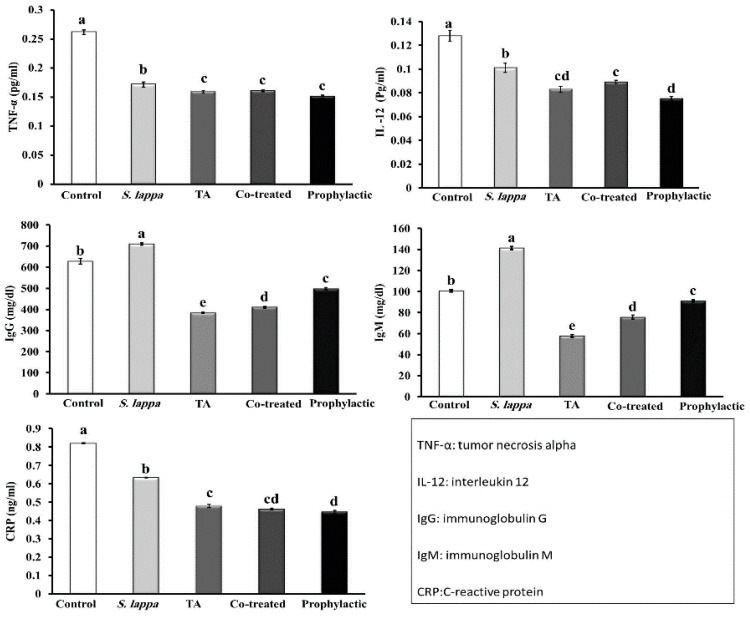
Effect of *Saussurea lappa* (*S. lappa*) and/or triamcinolone acetonide (TA) treatment on tumor necrosis factor-alpha (TNF-α), interleukin-12 (IL-12), immunoglobulins G and M (IgG and IgM), and C-reactive protein (CRP) concentrations in rats’ serum. A significant (*p* < 0.05) difference between groups is represented by different letters (a, b, c, and d) above the bars. Letter (a) clarifies the greatest value. The values are the means ± SE. (*n* = 5/group).

As shown in Figure 3, *S. lappa* ethanolic extract oral administration for three weeks significantly depressed the release of pro-inflammatory cytokines (IL-12, and TNF-α) and CRP relative to the control group. Moreover, a significant boost of the immunoglobulin levels (IgG and IgM) was evident in the *S. lappa* orally administered rats relative to the control group.

On the other hand, TA administered rats showed a significant reduction in IL-12, TNF-α, CRP, IgG, and IgM levels relative to the control group. In contrast, the *S. lappa* ethanolic extract application in both prophylactic and therapeutic groups significantly restored the levels of the depleted immunoglobulin. Still, it did not significantly alter IL-12 and TNF-α levels compared to the TA-only-exposed group. However, the *S. lappa* administration in the prophylactic group significantly reduced the CRP level relative to the TA-only-exposed group.

### 3.5. Tissue Antioxidative Status and Lipid Peroxidation

**Figure 4:**
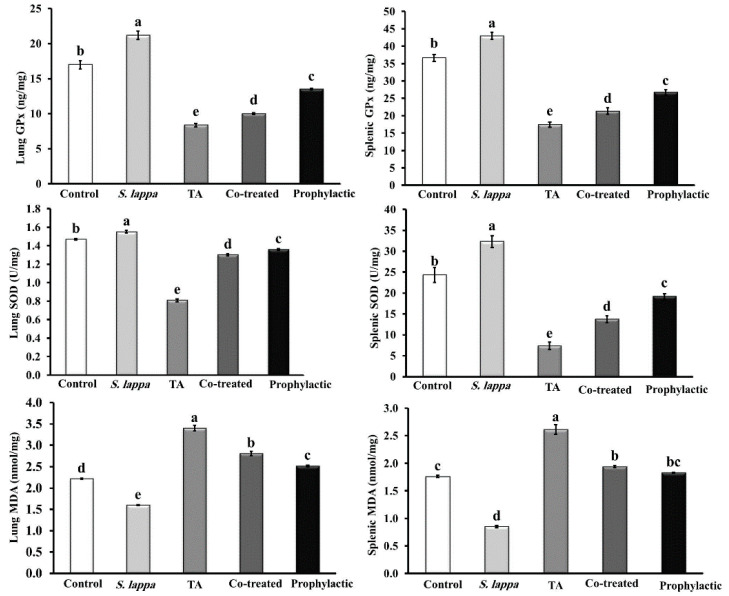
Effect of *Saussurea lappa* (*S. lappa*) and/or triamcinolone acetonide (TA) on the antioxidant/oxidant status of the lung and splenic tissues of rats. A significant (*p* < 0.05) difference between groups is represented by different letters (a, b, c, and d) above the bars. Letter (a) clarifies the greatest value. The values are the means ± SE. (*n* = 5/group).

As shown in Figure 4, dosing of normal animals with *S. lappa* ethanolic extracts for three weeks significantly (*p* < 0.05) increased the activities of antioxidant enzymes (SOD and GPx) in lung and splenic tissues compared to the control group. However, significant depression of the lipid peroxidation marker MDA was evident in *S. lappa*-treated rats relative to control rats.

However, intraperitoneal injection of rats with 40 mg TA/kg two times weekly for 14 days induced a significant (*p* < 0.05) reduction in the SOD and GPx content in lung and splenic tissues with an elevation of MDA concentration relative to the control group. Moreover, *S. lappa* showed a remarkable protective role against TA-induced oxidative stress through partial restoration of these enzyme levels near to the control values.

### 3.6. Histopathological Observation of Lung and Spleen

**Figure 5:**
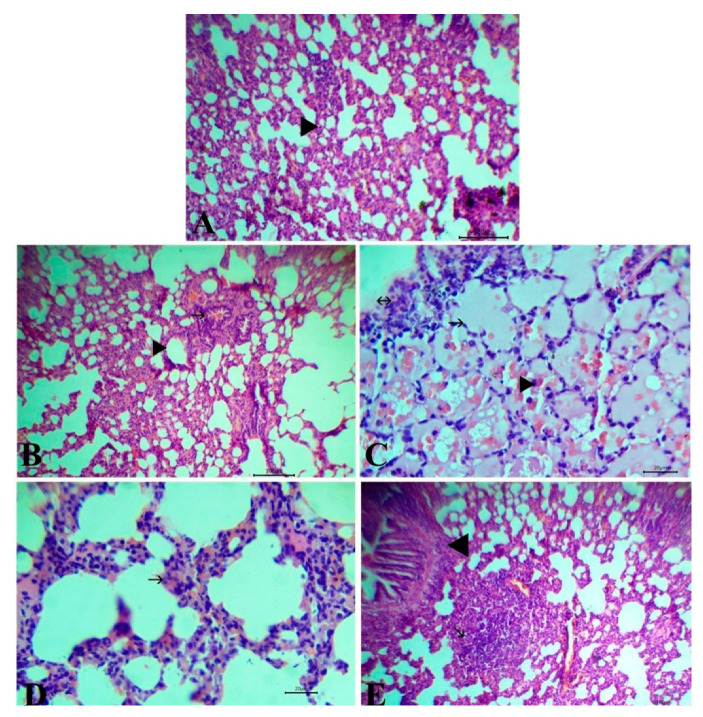
Representative photomicrographs of H&E stained lung tissue of control group (**A**) and the *Saussurea lappa*-alone-treated group (**B**) showing typical histological structure with alveoli (arrowhead) and bronchus (arrow), Bar 100. Rats injected with triamcinolone acetonide (TA) showing pulmonary edema (arrow) with congestion of perialvealar capillaries and mononuclear cells (arrowhead) with a focal aggregation of lymphocytes (arrow with two head) (**C**), Bar 20. The co-treated group showed mild thickening of interalveolar septa with red blood cells and a few mononuclear cells (arrow) (**D**), Bar 20. The prophylactic group showed aggregation of lymphocytes forming lymphoid follicles (arrow) (**E**), Bar 100. Magnification: 100×.

Microscopical examination of H&E stained lung sections of negative control and *S. lappa*-alone-treated rats showed normal bronchioles, alveoli, and blood vessels (Figure 5A,B). Meanwhile, rats injected with TA showed pulmonary edema represented by alveoli filled with serosanguinous fluid with congestion of perialvear capillaries and infiltration of mononuclear cells beside the focal aggregation of lymphocyte subpleural (Figure 5C). The lung section of the co-treated group exhibited mild thickening of interalvealar septa with RBCs and few mononuclear cells (Figure 5D), while rats in the prophylactic group manifested normal structure with the aggregation of lymphocytes forming lymphoid follicles as a defense mechanism (Figure 5E).

**Figure 6:**
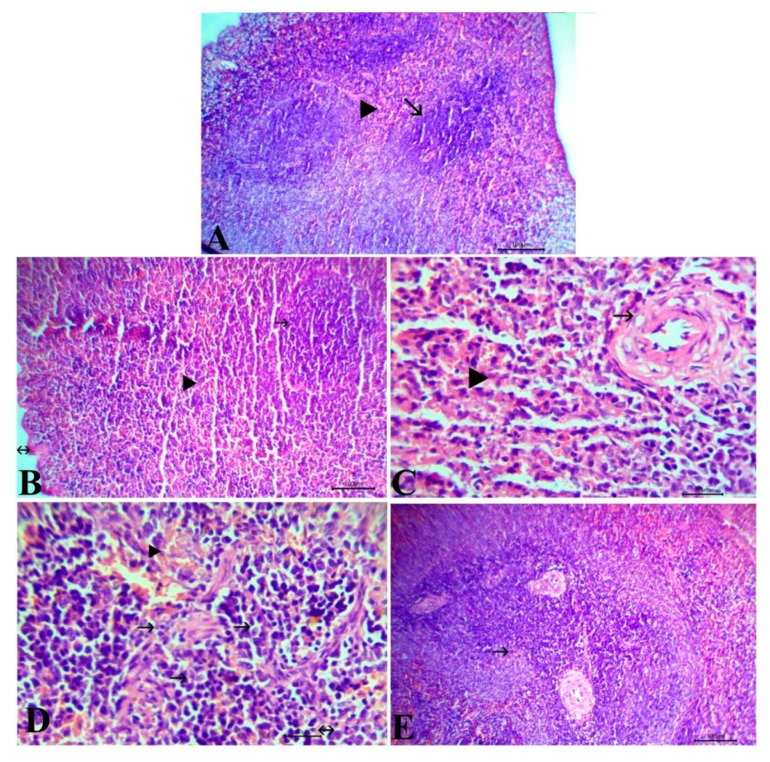
Representative photomicrographs of H&E stained spleen tissue of control group (**A**) and *Saussurea lappa*-alone-treated group (**B**) showing the normal histological structure, capsule (arrow with 2 head), white pulp (arrow), and red pulp (arrowhead), Bar 100. Triamcinolone acetonide-injected rats showing thickening, vacuolation, and endotheliosis in the wall of center arterioles (arrow), congestion of red pulp (arrowhead) (**C**), Bar 20. The co-exposed group showed mild hyperplasia of lymphocytes (arrow), a few infiltrations of mononuclear cells (arrow with two head) with congestion of the red pulp (arrowhead) (**D**), Bar 20. The prophylactic group showed hyperplasia of lymphocytes in the white pulp (arrow) (**E**), Bar 100. Magnification: 100×.

The control and *S. lappa*-treated groups revealed normal splenic tissue, including fibrous capsules from which trabeculae enter into the parenchyma-containing central arterioles in the center of white pulp surrounded with red pulp (Figure 6A,B). On the other hand, TA-injected rats showed thickening, vacuolation, and endotheliosis in the wall of the center arterioles. In addition, necrosis and depletion of lymphocytes inside the white pulp and reduction of the size of the lymphoid follicles in addition to congestion of the red pulp were observed (Figure 6C). The co-treated group exhibited mild hyperplasia of lymphocytes inside white pulp and a few infiltrations of mononuclear cells with congestion of the red pulp (Figure 6D). The rats that received *S. lappa* as protection in the prophylactic group showed hyperplasia of lymphocytes inside the white pulp (Figure 6E). All the detected lesions in the lung and spleen tissues of all experimental groups were scored and are displayed in Table 4.

### 3.7. Immunohistochemical Results

**Figure 7:**
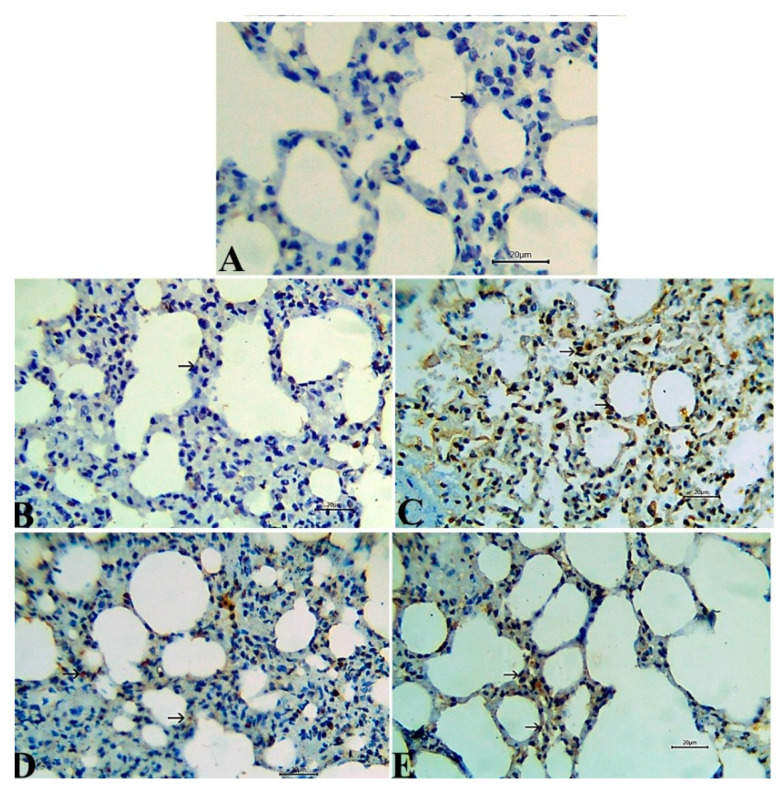
Representative photomicrographs of the lung tissue with immunoexpression of the Caspase-3 that showed a negative reaction in the rats of normal control and *Saussurea lappa*-only-treated groups (**A**,**B**), while the strong reaction was seen in triamcinolone acetonide-exposed rats (**C**). Moderate cytoplasmic reactivity was observed in the co-treated group. (**D**) Mild reactivity was recorded in prophylactic *S. lappa*-treated rats (**E**), Bar 20. Magnification: 400×.

**Figure 8:**
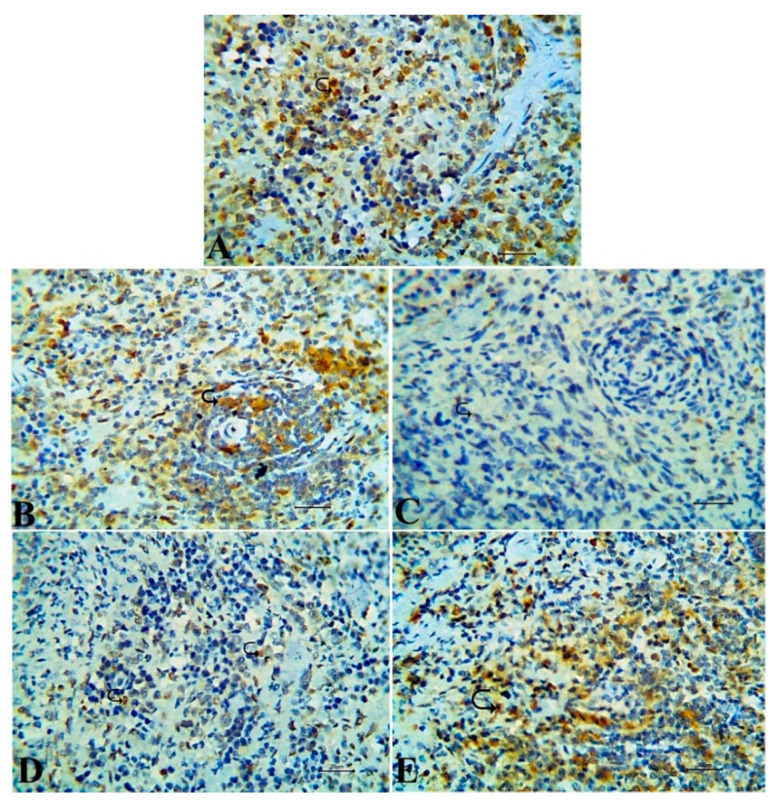
Representative photomicrographs of the splenic tissue with immunoexpression of CD8+ cells that showed strong a positive expression in the rats of normal control and *Saussurea lappa*-only-treated groups (**A**,**B**), while negative to weak expression was noticed in triamcinolone acetonide-injected rats (**C**). The co-treated group exhibited a mild degree of reaction (**D**); while a moderate degree of expression was seen in prophylactic *S. lappa*-treated rats (**E**), Bar 20. Magnification: 400×.

Caspase-3 immunoexpression, a marker for caspase-dependent apoptosis, was evaluated to investigate the underlying mechanism of TA on endothelial cells. The activity of the Caspase-3 enzyme in the lung tissue was augmented by TA administration, as observed in Figure 6C that was manifested by strong immunoreactivity compared to the rats in both control and *S. lappa*-alone-treated groups, which revealed an adverse reaction of this enzyme (Figure 7A,B). The Caspase-3 protein expression was reduced by *S. lappa* treatment of TA-injected rats, but not completely normalize (Figure 7D,E). CD8^+^ as a marker of activated T lymphocytes exhibited negative to weak expression in the splenic tissues of TA-only-treated rats Figure 8C, and strong positive expression in both healthy control and plant-alone-administered animals (Figure 8A,B). The number of CD8^+^ immune-positive cells was enhanced in the TA-exposed groups treated with *S. lappa* varying from a mild to moderate degree in both co-treated and prophylactic groups, respectively, (Figure 8D,E). The scores are summarized in Table 4.

## 4. Discussion

Currently, a growing global trend has been directed toward the use of natural products as prophylactic food supplements or therapeutic agents [50,51,52,53,54,55]. GCs are well-known, effective, anti-inflammatory agents. Nonetheless, their frequent use may potentiate destructive impacts on the body’s immune system. TA is a synthetic analog of hydrocortisone with very poor solubility in aqueous solutions and a low rate of diffusion into tissue and cellular layers resulting in longstanding and deleterious effects [26]. However, research on the treatment efficacy of TA and its side effects, particularly the effects on immune components, hematopoietic elements, and antioxidant systems, is still growing. Hence, this study tested, for the first time, the efficacy of *S. lappa* to rescue TA immunosuppression and oxidative stress and the concomitant influence on its anti-inflammatory action.

Initially, the erythrogram and leukogram components were not significantly altered in the *S. lappa* ethanolic-extract-treated group except for notable leukocytosis and lymphocytosis. A comparable hemogram was previously reported following *S. lappa* oral administration in rats [56] and rabbits [57]. The increment of lymphocyte count is usually associated with the enhancement of murine immunological function [58]. On the other hand, the hematological analysis presented here showed that the blood parameters were highly sensitive to TA administration, and they are principle indicators of immunity. A significant elevation in Hb content, RBCs count, and PCV value were recorded in the TA-treated group. The TA-altered erythrogram might be attributed to TA’s effect on mother cell differentiation and proliferation in the bone marrow with the enhancement of RBCs production rather than an increase in the survival rate of RBCs. In the same line, Tang et al. [59] indicated that the increase in RBC mass during GCs overexposure was accompanied by a significant up-regulation of erythropoietin hormone with subsequent stimulation of the erythropoiesis process. Besides, GCs might ultimately affect erythropoietin hormone transcription by modifying the expression of other transcription factors [60]. Our results are supported by the findings of Ohkaru et al. [61] who revealed that daily administration of dexamethasone (1mg/kg b.wt s/c) for ten days increased the number of RBCs, Hb content, and hematocrit value in rats through decreasing the extracellular volume. Exposure to many stress facets such as injury, infection, drugs, hormones, chemicals, and pollutants may affect the immune function at a cellular level through leukocyte redistribution that may be decreased or increased [62]. Therefore, the changes noticed in blood leukocyte numbers could be a useful marker of stress conditions. On the contrary, *S. lappa* oral administration markedly modulated the deviations in hemogram parameters, particularly in the prophylactic regime. Such improvement could be highly linked to the multiple bioactive actions of *S. lappa* with well-known hemopoietic activity and have a direct influence on the production of blood cells [37].

GC hormones are the key mediators of stress, and this was demonstrated here as TA-administered rats exhibited a higher TLC with neutrophilia. Still, a significant reduction in lymphocytes, monocytes, eosinophils, and basophils count was evident. In this regard, earlier studies verified that although GCs have an immunosuppressive effect on immune cells, they exert multiple and sometimes contradictory effects on neutrophils [63]. The observed neutrophilia after TA application may be owed to the enhanced survival rate of circulating neutrophils by overwhelming their apoptosis [64]. In addition, TA could delay neutrophils transmigration to the inflammatory sites via inhibition of leukocyte adhesion molecules [65]. Interestingly, despite the evident apoptotic activity of TA in lung tissue in the current study, the previous findings confirmed GCs’ ability to induce apoptosis in dendritic cells, eosinophils, monocytes, basophils, and T lymphocytes, while they strongly delay the rate of apoptosis in neutrophils [66,67,68]. Additionally, GCs increase granulocyte colony-stimulating factor, which consecutively alters the neutrophils proliferation [69]. Lymphopenia observed in the TA group may be an index of immunosuppression. In the same line, Grace et al. [70] detected leukocytosis in rats that received an I/P dose of dexamethasone (5mg/kg) for three days in comparison to the negative control. The authors assigned increments to the lowering actions of GCs on the immune system that make the body more susceptible to infections. In addition, Anafi et al. [71] showed that lymphopenia, monocytopenia, eosinopenia, in addition to neutrophilia were detected in rats exposed to high doses of dexamethasone. Importantly, *S. lappa* oral dosing significantly reversed the TA-induced neutrophilia and lymphocytopenia. A pronounced effect was evident in the prophylactic group. The favorable effect of the phenolic and flavonoids compounds in *S. lappa* treatment on blood cells production could be responsible for the adjustment of the different leukocytes count [37]. In addition, the anti-apoptotic activity of *S. lappa* detected here and in other types of cells in earlier studies [72,73] could partly share in suppressing neutrophil proliferation.

Herein, prominent anti-inflammatory and immunostimulant activities were recorded in rats that orally received *S. lappa* for three weeks. Importantly, dehydrocostuslactone, the major component of *S. lappa* extract as detected in GC-MS analysis, has been shown to have an anti-inflammatory effect linked with the inhibitions of STAT3 and NF-κB [74] and with heme oxygenase-1 induction [75]. In addition, dehydrocostuslactone inhibited lipopolysaccharide-induced inflammation by p38MAPK-dependent induction of hemeoxygenase-1 in an in vitro model, and it improved survival of cecal ligation in a puncture-induced mouse model of sepsis in vivo [76]. Similarly, the usage of 400 and 600 mg/kg b.wt *S. lappa* extract in arthritic rats reduced the serum levels of CRP, TNF-α, IL-6, and IL-1β [10]. Sesquiterpene lactone fraction of *S. lappa* inhibited the production of the TNF-α in murine macrophage-like cells [12,77]. On the other hand, Lim et al. [78] explored the anti-inflammatory effect of isolated alantolactone compounds from *S. lappa* through its ability to suppress IFN-γ- and TNF-α-induced production of IL-8 by blocking phosphorylation of signal transducers and activators of transcription in human culture cells.

In contrast, a significant drop in the serum levels of CRP, IL-12, TNF-α, IgG, and IgM indicated the capacity of TA to suppress the cells innate and adaptive immunity by inhibiting the synthesis of pro-inflammatory cytokines. CRP is an acute-phase protein generated in the liver, described as an inflammatory effector molecule. It may be one of the links between nonspecific innate immunity and specific clonal immunity because of its capacity to trigger the classical cascade that mediates phagocytosis and improves antigen presentation [79]. Moreover, IL-12 is a pro-inflammatory cytokine produced by monocytes and dendritic cells in concert with INFγ; it is essential to the development and differentiation of Th1-lymphocytes with the secretion of Th1 cytokines such as TNF-α and INFγ, so it is considered the link between cellular and innate immunity [80]. GCs block not only IL-12 secretion but also downregulate IL-12 R β1 and β2 expression on T cells. Such substantial GC-induced inhibition of the Th1 immune response may evoke severe cellular immunodeficiency and diminished defense to intracellular and opportunistic infections [81]. Recently, the in vitro study of Siebelt et al. [82] reported the downregulation action of TA on the inflammatory cascade through its stimulation of a specific macrophage subtype called regulatory macrophages, which are considered an anti-inflammatory form of M2-activated macrophages; also, TA decreased IL-10 production. In line with this observation, several investigators accounted for the immunosuppressive effect of GCs by their inhibition of immunoglobulin synthesis [83] and reduction of the production of complement system components [84]. Additionally, certain types of activated T lymphocytes and natural killer cells are susceptible to GC-induced apoptosis [85]. In addtion, the negative influence of TA on the immune system could be related to the inhibition of the appearance of some CDs of Ag antigens of dendritic cells [25]. Our findings agree with the studies of Kumari et al. [86] and Pashikanti et al. [87]. Suppression of serum immunoglobulin levels was confirmed microscopically by depletion of lymphocytes inside the white pulp of spleen with a reduction of the size of the lymphoid follicles in the TA-exposed group. In addition, a few sporadic CD8^+^ expressions in the immune-histochemical investigation were observed. CD8^+^ T-cells are a critical component of the cellular immune response. Correspondingly, Wang et al. [39] indicated that both helper T cells (CD4^+^) and cytotoxic (CD8^+^) populations were decreased in the blood of mice that were I/P injected with dexamethasone (25 mg/kg) for three days.

In contrast, significant restoration of the immunoglobulin titer was recorded in TA-exposed rats that received *S. lappa*. This immunostimulant activity of *S. lappa* may be ascribed to the several bioactive compounds present in the extract based on the GC-MS characterization, comprising dehydrocostus lactone, dihydrodehydrocostus lactone, caryophyllene oxide, saussurea lactone, costunolide, and beta-costol. Active sesquiterpenes lactones, including dehydrocostus lactone, dihydrodehydrocostus lactone, and costunolide, were formerly isolated from *S. lappa* methanolic extracts. These bioactives have been known to have beneficial immunomodulatory activities. For instance, dehydrocostuslactone and costunolide showed their immunomodulatory actions through inhibiting the killing activity of cytotoxic T lymphocytes by inhibiting the rise in tyrosine phosphorylation in response to the cross-linking of T cell receptors [76]. These findings are corroborative by the results of Pandey [37] that reported enhancement of antibody titer with elevated TLC and spleen weight when using a high dose of *S. lappa* hydroethanolic extract in mice. Regarding the outcome of *S. lappa* administration on TA anti-inflammatory activity, in the *S. lappa* prophylactic group, there was a nonsignificant trend toward decreased release of the pro-inflammatory cytokines TNF-alpha and IL-12. However, a significant reduction in the CRP level was apparent in the prophylactic group compared to the TA-only-treated group. These results could suggest that *S. lappa* administration could potentiate the TA anti-inflammatory activity to a moderate extent in the prophylactic application. Similarly, another plant compound named Ginsenoside Rh1 potentiated dexamethasone’s anti-inflammatory effects in a collagen-induced arthritis mouse model [88].

In the current study, the oral administration of *S. lappa* ethanolic extracts for three weeks significantly augmented the activities of antioxidant enzymes (SOD and GPx) in lung and splenic tissues but depressed MDA accumulation, a lipid peroxidation biomarker. These results are in harmony with the results of Abdel-Rahman et al. [89]. In this regard, Sathuvan et al. [90] confirmed the presence of two major polysaccharides (SLT-3, SLT-4) in *Costus pictus*, which belongs to the same family as *S. lappa* (Costaceae), that efficiently prevented ROS generation, regulated GPx activities, and inhibited MDA formation. In addition, Benedetto et al. [91] demonstrated the ability of *S. lappa* to donate electrons to reactive radicals, converting them into more stable and unreactive species.

Conversely, a significant decrease in the concentrations of SOD and GPx activities but an increment in the MDA level was evident in both lung and splenic tissues of animals exposed to TA. Such changes could be a result of enhanced radicals generation during TA metabolism that overwhelms the natural antioxidant system in the body leading to oxidative damage and cell injury. These findings are compatible with Yi et al. [92], who revealed higher content of MDA in the spleen of mice I/P injected with dexamethasone. In addition, in the same study, suppression of SOD and GPx activities, as well as the presence of numerous apoptotic and necrotic cells, were reported. Current histopathological findings were very indicative of the oxidative stress- and lipid peroxidation-inductive nature of TA. Earlier studies mentioned that the excess GCs use usually resulted in the overproduction of ROS, which causes mitochondrial breaks, diminishes cellular vitality, and alters the cell permeability causing cell apoptosis [93].

Interestingly, the *S. lappa* pre- or concurrent administration in TA-exposed rats significantly reversed the restored antioxidant enzyme activity and suppressed the lipid peroxidation. In addition, activities of CAT, SOD, and GST enzymes were enhanced in deltamethrin-intoxicated animals fortified with aqueous extracts of *S. lappa* (300 mg) [94]. Saleem et al. [95] found that the protective effects of *S. lappa* extract offered significant safeguards against toxicant-induced depletion of marker enzymes and oxidative stress to an extent similar to that of the standard natural antioxidant α-tocopherol. This effect may possibly be related to the decrease of membrane damage and membrane fluidity because of its high content of flavonoids, phenolic, steroids, and chlorogenic acid [96]. In addition, *S. lappa’s* major bioactives, such as dehydrocostuslactone and costunolide, have been reported to play some crucial roles as antioxidant agents via conjugation with target proteins mercapto (SH)-groups to intervene in several main biological activities in cells [97,98]. The decreased MDA concentrations in the *S. lappa*-treated group may be due to their protective effect from the harmful effects of ROS-mediated lipid peroxidation of tissue macromolecules [99].

The antitumor activity of *S. lappa* and its major constituent dehydrocostus lactone have been studied in various types of cancers such as neuroblastoma [100], hepatocellular carcinoma [101], leukemia [102], lung cancer [103], and prostate cancer [104]. The plant and its bioactives showed a significant anticancer activity by triggering the apoptotic death of cancer cells. However, it seems that *S. lappa* has a different mode of action toward normal cells. For instance, in the study of Zhao et al. [73], *S. lappa’s* main bioactives significantly reduced the Caspase-dependent apoptotic pathway in neuronal cells, probably linked to the free radical scavenging activity. Besides, other natural products showed selective apoptotic activity [105,106]. For instance, dandelion root extract induced apoptosis in human pancreatic cancer cells with no significant effect on noncancerous cells [107]. In the current study, a weak Caspase-3 immunolabelling was detected in the lung sections of *S. lappa*-treated rats, suggesting its anti-apoptotic activity. Caspase-3 is one of the key executioners of apoptosis, capable of cleaving or degrading many key nuclear proteins [108]. Additionally, Caspase-3 immunoexpression has been used as a reliable tool to assess apoptotic activity in various earlier studies [109,110]. Thus, more investigation of the *S. lappa* apoptotic activity in normal cells could provide a basis on which further research in cancer treatment through *S. lappa* can be executed.

On the other hand, overexpression of Caspase- 3 as an apoptotic marker in the lung tissue of the TA-administered group was confirmed microscopically in the current study. This is in agreement with Harada et al. [31] who showed that treatment of cell culture with a high TA dose (1 mg/mL) caused irretrievable damage to cell viability and morphology and increased the number of apoptotic cells with higher mRNA expression of Caspase 3, 7, 8, and 9. In the same line, the classic component of GC-induced apoptosis includes invigoration of the intrinsic apoptotic pathway that occurs in the mitochondria in reaction to numerous stimuli such as GCs. GCs signaling elevates pro-apoptotic Bcl-2 expression, which can trigger the master-apoptotic proteins Bax/Bak to disturb mitochondrial membrane potential and, consequently, discharge cytochrome c and other apoptogenic proteins. This results in Caspase 9 activation and consequent effector Caspase 3 activation [111]. However, the *S. lappa* pre- or concurrent administration in TA-exposed rats significantly suppressed Caspase- 3 immunoexpression, which could be linked to their antioxidant activity.

Collectively, the oxidative stress and immunosuppression induced by TA were accompanied by a remarkable decline in body weight gain, which is clear from the significant reduction in the spleen and lung indexes recorded in this group. These findings are in harmony with the previous studies of Kumari et al. [86] and Lingaiah et al. [112]. Such reductions in the bodyweight could be elucidated based on the negative effects of GCs on the gut. GCs have been reported to reduce the non-stretched part of the small intestine and considerably reduce protein synthesis in the smooth muscle of the small intestine, which might lead to reduced food intake and body weight loss [113]. Administration of *S. lappa* extracts either before or in combination with TA administration significantly counteracted TA-evoked weight reduction. Such improvement could be the consequence of the improvement of the immune functions and antioxidant activities in rats.

Importantly, the safety profile of any herbal medicine is an important factor that should be considered to determine its therapeutic utility [114]. In this regard, Garg et al. [115] determined LD_50_ of *S. lappa* in Swiss Albino mice to establish its safety margin and verified that no death or signs of toxicity were observed in animals treated with the extract up to doses of 2000 mg/kg b.wt., which established its safety for use in further pharmacological screening. Although the published evidence to date and traditional use in various countries support the safety of *S. lappa,* more investigations are warranted on bioavailability, pharmacokinetics, physiological pathways, and the importance to human health [37].

## 5. Conclusions

Taken together, the combined biochemical, histopathological, and immunohistochemical results suggested that the *S. lappa* oral administration could efficiently rescue TA-induced immunosuppression. In addition, *S. lappa* could moderately augment TA-anti-inflammatory activity. Additionally, *S. lappa* could efficiently combat the TA-induced oxidative stress and apoptotic consequences in lung tissues. In addtion, the reclamation from the baleful effects of TA is highly dependent on the method of *S. lappa* supplementation. *S. lappa* application as a prophylactic regime achieved better results than as a therapeutic one.

## Figures and Tables

**Table 1:** Retention time (RT) and peak area (%) of the different compounds found in *Saussurea lappa* ethanolic extract analyzed by GC-MS.

Compound	RT (min)	Peak Area %	Molecular Formula	Molecular Weight
Dehydrocostuslactone	30.41	49.68	C_15_H_18_O_2_	230
Dihydrodehydrocostus lactone	29.21	16.34	C_15_H_20_O_2_	232
Caryophyllene oxide	27.02	11.03	C_15_H_24_O	220
Saussurea lactone	23.26	4.16	C_15_H_22_O_2_	234
Costunolide	26.31	3.59	C_15_H_20_O_2_	232
Beta-costol	25.55	2.74	C_15_H_24_O	220
(+)-Isovalencenol	31.71	2.52	C_15_H_24_O	220
Linoleic acid, methyl ester	32.1	2.15	C_19_H_34_O_2_	294
9-Octadecenoic acid, methyl ester	32.22	1.54	C_19_H_36_O_2_	296
Aristol-1(10)-en-9-ol	25.67	1.41	C_15_H_24_O	220
Hexadecanoic acid,methyl ester	28.89	0.85	C_17_H_34_O_2_	270
Linolenic acid, methyl ester	29.59	0.54	C_19_H_32_O_2_	292
Valerenol	30.64	0.46	C_15_H_24_O	220
Reynosin	31.05	0.39	C_15_H_20_O_3_	248
Beta-caryophyllene oxide	21.4	0.38	C_15_H_24_O	220
Linolein, 2-mono-	19.07	0.38	C_21_H_38_O_4_	354
10-Heptadecen-8-ynoic acid, methyl ester, (E)-	32.44	0.25	C_18_H_30_O_2_	278
Octadecanoic acid, methyl ester	32.71	0.24	C_19_H_38_O_2_	298
16-Methyloxacyclohexadeca-3,5-dien-2-one	32.81	0.24	C_16_H_26_O_2_	250
Santamarine	28.43	0.22	C_15_H_20_O_3_	248
Glycidyl oleate	33.39	0.22	C_21_H_38_O_3_	338
Santamarine	38.59	0.18	C_15_H_20_O_3_	248
Farnesene epoxide, E-	33.49	0.17	C_15_H_24_O	220
Linolein, 2-mono	17.61	0.16	C_21_H_38_O_4_	354
Eudesm-4(14)-en-11-ol	20.62	0.16	C_15_H_26_O	222
Trans-á-Ionone	38.52	0.15	C_13_H_20_O	192

**Table 2:** Effects of *Saussurea lappa* (*S. lappa*) and/or triamcinolone acetonide (TA) on body weight, body weight gain, and organ indexes of treated rats.

Parameters	Experimental Groups				
	Control	*S. Lappa*	TA	Co-Treated	Prophylactic
Initial body weight (g)	154.67 ± 3.18 ^a^	158.00 ±1.53 ^a^	157 ± 1.53 ^a^	157.67 ± 2.73 ^a^	157.33 ± 1.45 ^a^
Final body weight (g)	173.3 ± 7.26 ^a^	178.33 ± 4.41 ^a^	130 ± 0.58 ^c^	144.00 ± 3.06 ^b^	149.33 ± 0.88 ^b^
Body weight gain (g)	18.67 ± 4.10 ^a^	20.33 ± 3.18 ^a^	-25.67 ± 0.67 ^c^	−13.67 ± 0.33 ^b^	−8.00 ± 0.58 ^b^
Lung weight (mg)	1500 ± 11.55 ^a^	1506.67 ± 6.67 ^a^	913.3 ± 8.82 ^d^	1133.3 ± 35.28 ^c^	1225 ± 18.93 ^b^
Lung index (mg/g)	8.68 ± 0.33 ^a^	8.45 ± 0.19 ^ab^	7.02 ± 0.10 ^c^	7.86 ± 0.08 ^b^	8.07 ± 0.05 ^b^
Spleen weight (mg)	530 ±11.55 ^a^	520.00 ± 11.55 ^a^	303.33 ±12.02 ^d^	366.67 ±14.53 ^c^	420.00 ±17.32 ^b^
Spleen index (mg/g)	3.06 ± 0.07 ^a^	2.93 ± 0.08 ^a^	2.32 ± 0.11 ^c^	2.56 ± 0.10 ^bc^	2.81 ± 0.13 ^ab^

The data are expressed as the mean ± SE. Means within the same row carrying different superscripts (a, b, and c) are significantly different (one-way ANOVA followed by Duncan’s multiple range test, *p* < 0.05).

**Table 3:** Effects of oral dosing of *Saussurea lappa* (*S. lappa*) and/or triamcinolone acetonide (TA) on hematologic variables of treated rats.

Parameters	Experimental Groups				
	Control	*S. Lappa*	TA	Co-Treated	Prophylactic
RBCs (× 10^6^/µL)	6.92 ± 0.12 ^d^	6.84 ± 0.21 ^d^	10.77 ± 0.20 ^a^	8.69 ± 0.26 ^b^	7.66 ± 0.39 ^cd^
Hb (gm %)	14.47 ± 0.67 ^d^	14.83 ± 0.36 ^d^	21.40 ± 0.46 ^a^	17.93 ± 0.35 ^b^	16.35 ± 0.26 ^c^
PCV (%)	43.58 ± 0.98 ^d^	44.17 ± 0.52 ^d^	64.60 ± 1.18 ^a^	56.24 ± 1.19 ^b^	49.00 ± 1.10 ^c^
TLC (103/µL)	10.87 ± 0.15 ^d^	15.68 ± 0.64 ^a^	16.34 ± 0.45 ^a^	14.24 ± 0.20 ^b^	12.78 ± 0.21 ^c^
Neutrophil (10^3^/µL)	2.14 ± 0.05 ^d^	2.25 ± 0.10 ^d^	10.38 ± 0.28 ^a^	7.86 ± 0.16 ^b^	5.37 ± 0.25 ^c^
Lymphocyte (10^3^/µL)	8.30 ± 0.18 ^b^	13.00 ± 0.56 ^a^	5.69 ± 0.28 ^d^	6.05 ± 0.10 ^cd^	7.02 ± 0.06 ^c^
Monocyte (10^3^/µL)	0.24 ± 0.01 ^ab^	0.25 ± 0.02 ^a^	0.14 ± 0.01 ^d^	0.18 ± 0.01 ^c^	0.21 ± 0.01 ^b^
Eosinophil (10^3^/µL)	0.17 ± 0.01 ^ab^	0.18 ± 0.01 ^a^	0.11 ± 0.01 ^d^	0.13 ± 0.01 ^c^	0.15 ± 0.00 ^b^
Basophil (10^3^/µL)	0.02 ± 0.001 ^a^	0.02 ± 0.003 ^a^	0.01 ± 0.001 ^c^	0.01 ± 0.001 ^bc^	0.02 ± 0.001 ^ab^

The data are expressed as the mean ± SE. Means within the same row carrying different superscripts (a, b, and c) are significantly different (one-way ANOVA followed by Duncan’s multiple range test, *p* < 0.05).

**Table 4:** Histopathological lesion scores of the lung and spleen in different experimental groups.

Organs	Descriptive Lesions	Control	*S. Lappa*	TA	Co-Treated	Prophylactic
Spleen	Thickening in the wall of center arterioles	−	−	+++	−	−
	Congestion of red pulp with inflammatory cells infiltration	−	−	+++	+	−
	Depletion of lymphocytes inside white pulp	−	−	+++	−	−
	Hyperplasia of white pulp	−	−	−	+	++
	Splenic CD8^+^	++++ (51%–75%)	++++ (51%–75%)	+ (<10%)	++ (10%–25%)	+++ (26%–50%)
Lung	Pulmonary edema	−	−	+++	−	−
	Thickening of interalvealar septa	−	−	+++	+	−
	A focal area of necrosis	−	−	+++	−	−
	Aggregation of lymphocytes to form lymphoid follicles	−	−	−	+	++
	Lung Caspase-3	+ (<10%)	+ (<10%)	++++ (51%–75%)	+++ (26%–50%)	++ (10%–25%)

Lesions were scored for severity (− absent, + mild, ++ moderate, +++ severe). *Saussurea lappa* (*S. lappa*), triamcinolone acetonide (TA).

## Abbreviations

CRP	C-reactive protein
GC–MS	gas chromatography/mass spectrometry analysis
GCs	glucocorticoids
GPx	glutathione peroxidase
IgG	immunoglobulins G
IgM	immunoglobulins M
IL-12	interleukin-12
MDA	malondialdehyde
NO	nitric oxide
ROS	reactive oxygen species
SOD	superoxide dismutase
TA	triamcinolone acetonide
TNF-α	tumor necrosis factor-alpha
